# Pattern-Matched Powered Gait Orthosis Training in Patients with Neurological Gait Impairment: A Multicenter Prospective Pilot Study of Hip and Knee–Ankle–Foot Orthoses

**DOI:** 10.3390/jcm15103580

**Published:** 2026-05-07

**Authors:** Yeo Joon Yun, Changwon Moon, Ki-Hoon Kim, Tae-Hoon Kim, Bo-Kyoung Kim, HyukJae Choi, Dongbin Shin, Hyeyoun Jang, Seong Ho Jang, Mi Jung Kim

**Affiliations:** 1 Department of Physical Medicine and Rehabilitation, Hanyang University Guri Hospital, Hanyang University College of Medicine, 153 Gyeongchun-ro, Guri 11923, Republic of Korea; yunyujun@naver.com (Y.J.Y.);; 2 Department of Physical Medicine and Rehabilitation, Chungnam National University Hospital, Chungnam National University College of Medicine, 282 Munhwa-ro, Jung-gu, Daejeon 35015, Republic of Korea; 3 Department of Physical Medicine and Rehabilitation, Korea University Anam Hospital, Korea University College of Medicine, 73 Inchon-ro, Seongbuk-gu, Seoul 02841, Republic of Korea; 4 Department of Physical Medicine and Rehabilitation, Hanyang University Seoul Hospital, Hanyang University College of Medicine, 222-1 Wangsimni-ro, Seongdong-gu, Seoul 04763, Republic of Korea; 5 Seongdong Rehabilitation Clinic, 233 Haengdang-ro, Seongdong-gu, Seoul 04757, Republic of Korea; 6 Rehabilitation Engineering Research Institute, Korea Workers’ Compensation & Welfare Service, 17 Jamsil-ro, Songpa-gu, Seoul 05510, Republic of Korea; choi4215@naver.com; 7 Hexar Humancare Co., Ltd., 109 Jungang-ro, Danwon-gu, Ansan 15439, Republic of Korea

**Keywords:** powered gait orthosis, hip orthosis, knee–ankle–foot orthosis, neurological gait impairment, gait rehabilitation, multicenter pilot study

## Abstract

**Background**: Wearable powered gait orthoses offer a clinically flexible alternative to treadmill-based robotic systems, yet evidence on different device configurations matched to the site of neuromuscular impairment remains limited. **Methods**: In this multicenter prospective pilot study, 75 participants with neurological gait impairment were allocated to a hip orthosis (HO; *n* = 39) or a knee–ankle–foot orthosis (KAFO; *n* = 36) group based on clinical assessment of predominant gait pattern. Both groups completed six overground gait-training sessions over three weeks. Primary outcomes were the Six-Minute Walk Test (6MWT) and Ten-Meter Walk Test (10MWT), assessed without (WO) and with (WITH) the device. Secondary outcomes were the Berg Balance Scale (BBS), Timed Up and Go Test (TUG), and Dynamic Gait Index (DGI), all assessed without the device. Wilcoxon signed-rank tests were used for pre-to-post comparisons. **Results**: Both groups demonstrated significant improvements in primary walking outcomes, with consistent gains in unassisted (WO) 6MWT and 10MWT performance across groups and in device-assisted (WITH) 10MWT speed; the one exception was a small statistically significant but clinically negligible decrease in device-assisted 6MWT in the KAFO group (−4.1 m, below established MCID). In the KAFO group, BBS improved by a median of 5.5 points (43.5 to 49.0, *p* = 0.0005), TUG decreased by 5.1 s (*p* < 0.001), and DGI improved by 6.0 points (*p* = 0.002); all three changes exceeded published minimum detectable change thresholds. In the HO group, pre-to-post differences in BBS (+1.0), TUG (+0.8 s; an unfavorable direction), and DGI (−2.0; an unfavorable direction) were statistically detectable but small in absolute magnitude, fell at or below published thresholds for minimum detectable change, and should not be interpreted as clinically meaningful improvement. The WO-WITH performance gap in the KAFO group narrowed substantially after training, with 10MWT time no longer differing significantly between conditions at post-training (*p* = 0.116). **Conclusions**: Six sessions of gait pattern-matched powered gait orthosis training produced clinically meaningful within-group improvements in walking outcomes in both groups. In the KAFO group, balance and functional mobility outcomes also showed clinically meaningful improvements; in the HO group, balance and functional mobility outcomes showed only statistically detectable but clinically non-meaningful fluctuations around near-ceiling baseline scores. Walking benefits generalized to unassisted ambulation in both groups. These findings support the feasibility of an individualized orthosis prescription framework and provide a basis for future randomized controlled trials.

## 1. Introduction

Neurological gait impairment is a common and debilitating consequence of central nervous system disorders, including stroke, traumatic brain injury, and neurodegenerative conditions [1,2]. The recovery of walking function is consistently identified as one of the highest priorities among patients undergoing rehabilitation, as gait disability profoundly affects independence, community participation, and overall quality of life [3,4]. Consequently, developing effective and accessible gait rehabilitation strategies remains a central focus of neurorehabilitation research.

In recent years, robot-assisted gait training (RAGT) has gained considerable attention as a complementary approach to conventional physiotherapy [5,6,7]. Systematic reviews and meta-analyses have demonstrated that RAGT can improve walking speed, endurance, and functional mobility in patients with stroke and other neurological conditions [8,9,10]. However, the majority of evidence has been generated using large, stationary exoskeletons that are tethered to treadmill-based systems or require specialized institutional settings [11,12]. While these devices provide highly controlled training environments, their size, cost, and limited portability restrict their clinical applicability [13,14].

To address these limitations, wearable powered gait orthoses have emerged as a promising alternative that enables overground gait training in more flexible clinical settings [15,16]. Unlike stationary systems, wearable orthoses allow patients to practice walking in more ecologically valid environments while still receiving powered assistance [17]. Recent clinical trials have demonstrated the feasibility and preliminary effectiveness of wearable hip exoskeletons and knee–ankle–foot orthoses in improving gait parameters in neurological populations [18,19,20]. However, most studies to date have evaluated a single device type, and there is a notable gap in the literature regarding the within-group changes associated with different orthosis configurations tailored to the specific site of neuromuscular impairment.

From a clinical perspective, patients with neurological gait impairment present with heterogeneous gait patterns that reflect varying degrees of dysfunction at the hip, knee, and ankle [20,21]. This variability suggests that a one-size-fits-all approach to powered orthosis prescription may not be optimal. Instead, an individualized, gait pattern-matched strategy that selects a device specifically addressing the patient’s predominant gait deficit may yield superior functional outcomes. Our group previously reported preliminary findings on a powered Hip–Knee–Ankle–Foot Orthosis (HKAFO) in a single-arm pilot of chronic stroke patients, demonstrating short-term improvements in balance and device-assisted gait over six training sessions [22]. To our knowledge, no multicenter study has prospectively evaluated two distinct powered orthosis types within a gait pattern-matched allocation framework.

The present study aimed to characterize the within-group changes associated with a short-term powered gait orthosis training program using two pattern-matched orthoses: a hip orthosis (HO) targeting hip-driven gait deficits, and a knee–ankle–foot orthosis (KAFO) targeting knee and ankle gait deficits. We hypothesized that both orthoses would improve gait function after a three-week training program, and that the pattern of improvement would differ between the two groups in accordance with each device’s biomechanical target.

## 2. Materials and Methods

### 2.1. Study Design

This study was designed as a prospective, multicenter, open-label, pre–post comparative pilot study. Participants were enrolled across four institutions: Hanyang University Seoul Hospital, Korea University Anam Hostipal, Chungnam National University Hostiptal, and Seongdong Rehabilitation Clinic. The study protocol was approved by the Institutional Review Board of Hanyang University Medical Center (IRB No. HYUIRB-202304-025) and conducted in accordance with the ethical principles of the Declaration of Helsinki. This study was registered at the Clinical Research Information Service (CRIS, https://cris.nih.go.kr (accessed on 5 May 2026)) under registration number KCT0011412. Participant recruitment was conducted between 12 May 2023 and 12 November 2024. Written informed consent was obtained from all participants prior to enrollment.

**Figure 1:**
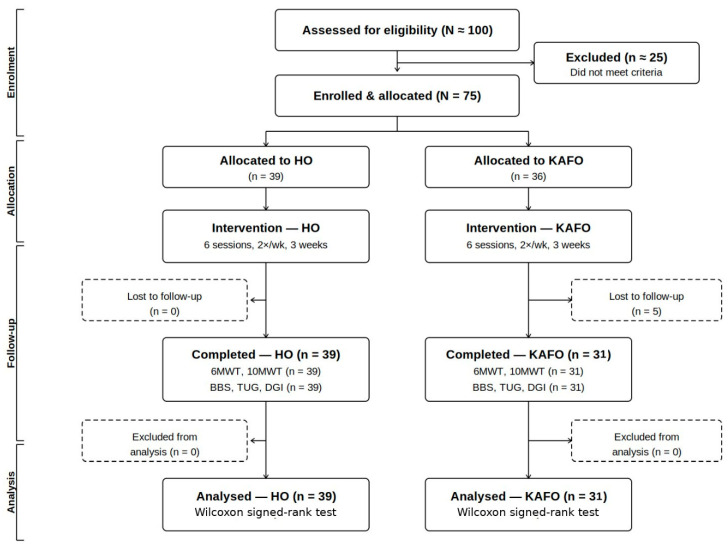
A CONSORT flow diagram of participant enrolment, allocation, follow-up, and analysis. Participants were allocated to the hip orthosis (HO) group or the knee–ankle–foot orthosis (KAFO) group based on clinical assessment of lower limb impairment. Five participants in the KAFO group were lost to follow-up after allocation, resulting in *n* = 31 for post-training assessments in that group. All 39 participants in the HO group completed the protocol. Dashed boxes indicate dropout and exclusion from analysis. HO = hip orthosis; KAFO = knee–ankle–foot orthosis; 6MWT = Six-Minute Walk Test; 10MWT = Ten-Meter Walk Test; BBS = Berg Balance Scale; TUG = Timed Up and Go Test; DGI = Dynamic Gait Index.

Participants were allocated to one of two device groups based on standardized clinical observation by an experienced physiatrist at each of the four participating sites prior to enrollment, independent of outcome assessment. The supervising physiatrist identified the predominant gait deficit pattern (hip-driven versus knee- or ankle-pattern deficits) and matched the participant to the orthosis with the corresponding biomechanical action. The assessment relied on routine clinical pattern recognition by board-certified rehabilitation physicians and was not based on a quantitative scoring instrument, formal video review, or a pre-specified algorithmic checklist; this reflects the pragmatic clinical-routine nature of the pilot design. To support consistency across sites, a referral pathway was pre-specified whereby any ambiguous case could be discussed with a physiatrist from another participating institution before final allocation; in practice, no such ambiguous case arose during recruitment, and all allocations were made by the original site assessor. Although baseline lower limb manual muscle testing scores and several other clinical–functional indices did not differ markedly between the resulting groups, allocation was guided by the kinematic pattern of gait deficit rather than by overall motor impairment severity, consistent with the device-pattern matching rationale of the study. Those presenting with predominantly hip-driven gait deficits, such as reduced hip flexion during swing, diminished hip extension at push-off, or hip-predominant compensatory gait strategies, were assigned to the HO group. Those presenting with knee instability during stance, inadequate knee flexion during swing, or foot drop were assigned to the KAFO group. The participant flow is presented in Figure 1.

### 2.2. Participants

A total of 75 participants with neurological gait impairment were enrolled across the four sites. Eligible participants were adults aged 18 years or older with a confirmed diagnosis of a central nervous system disorder causing persistent lower limb weakness and gait impairment. Inclusion criteria were as follows: (1) a Functional Ambulation Category (FAC) score of 2 to 4; (2) the ability to follow at least two steps of a three-step verbal command; (3) sufficient upper limb function to use an assistive device such as a cane or crutches; (4) adequate passive range of motion in the hip, knee, and ankle joints permitting device application; and (5) medical stability sufficient to engage in at least one hour of supervised gait training per day. The contralesional or less-affected limb was required to have a Manual Muscle Testing (MMT) grade of at least 3, and spasticity assessed by the Modified Ashworth Scale (MAS) was required to be grade 2 or below at the target joints.

Exclusion criteria included (1) physical characteristics preventing proper device fitting; (2) implantable cardiac devices; (3) medically or neurologically unstable conditions such as uncontrolled seizures or severe ataxia; (4) significant cognitive or communicative impairment precluding safe participation; (5) uncontrolled comorbidities; (6) severe lower limb deformities or recent fractures; and (7) any condition deemed unsuitable by the site investigator.

### 2.3. Devices

**Figure 2:**
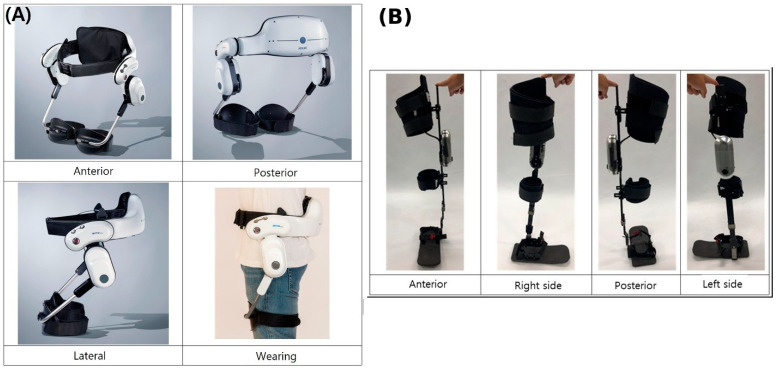
Photographs of the two powered gait orthoses used in this study. (**A**) Hip orthosis (HO): anterior, posterior, lateral, and wearing views. The HO is a bilateral powered hip orthosis incorporating brushless DC motors and harmonic gear reducers at the hip joints, mounted on a rigid lumbar frame with custom pelvic and thigh cuffs. (**B**) Knee–ankle–foot orthosis (KAFO): anterior, right side, posterior, and left side views. The KAFO is a unilateral powered orthosis with an active knee actuator, custom thigh and shank uprights, and an ankle–foot component, with the battery and controller unit mounted at the waist.

Two powered gait orthoses were used, both developed by Hexar Humancare Co., Ltd. (Ansan, Republic of Korea). Each device is classified as a wearable assistive robot for lower limb paralysis under Korean assistive technology regulations. Device selection was based on the predominant site of neuromuscular impairment as described in Section 2.1. Representative photographs of both devices are presented in Figure 2.

#### 2.3.1. Hip Orthosis (HO)

The HO is a wearable bilateral powered hip orthosis designed to assist patients with hip joint weakness or hip-predominant gait impairment. It comprises bilateral hip actuator modules, each incorporating a brushless DC (BLDC) motor, harmonic gear reducer, and absolute encoder, mounted on a rigid lumbar frame that also houses the controller and rechargeable battery (approximately 2 h of continuous use). Custom-fitted pelvic and thigh cuffs, fabricated by a certified orthotist, provide individualized body contact and force transmission. Gait-phase estimation derived from real-time hip joint kinematics drives phase-appropriate torque delivery: flexion assistance during swing to facilitate limb advancement, and extension support during late stance to promote push-off. Maximum torque output is 20 Nm per joint. The device supports level-ground and inclined walking, and resistive torque is applied during stair descent to reduce fall risk [17].

#### 2.3.2. Knee–Ankle–Foot Orthosis (KAFO)

The KAFO is a wearable powered knee–ankle–foot orthosis designed for patients with knee and ankle joint weakness or knee- and ankle-predominant gait impairment. Unlike conventional passive KAFOs that lock the knee in extension throughout the gait cycle, the powered KAFO provides active knee motion via a motorized actuator comprising a BLDC motor, a harmonic gear reducer, and pulley-belt transmission, mounted on the lateral knee upright. An inertial measurement unit (IMU) integrated in the thigh segment and an absolute knee encoder provide real-time gait-phase detection. A waist-mounted battery and controller unit supplies power, and custom thigh, shank, and ankle components are fabricated individually by a certified orthotist. Based on the estimated gait phase, the controller delivers active knee flexion assistance during swing, replicating the approximately 60° knee flexion arc of normal gait to improve foot clearance and step symmetry, and resistive torque during stance to prevent knee buckling. Maximum torque output is 20 Nm. A Bluetooth 5.0 module enables wireless real-time monitoring and parameter adjustment.

### 2.4. Intervention

Participants in both groups completed six overground gait-training sessions with their assigned device, conducted twice per week for three consecutive weeks (40 min per session; 240 min total). Sessions were conducted at the participant’s assigned clinical site under continuous supervision by at least one trained researcher or physical therapist.

Each session was structured as follows: (i) five-minute device fitting check and familiarization; (ii) two 10 m walk test (10MWT) trials as structured gait practice; (iii) one six-minute walk test (6MWT) trial; (iv) approximately 10 min of seated rest; and (v) a second 6MWT trial. The within-session walking bouts served as structured practice and were not used for formal outcome scoring. An adjustable suspension harness was used throughout all sessions for safety. Assistive torque levels and walking cadence were titrated by the supervising clinician based on participant tolerance and real-time device readouts. All parameter adjustments, rest periods, and adverse symptoms were documented in standardized case report forms. During the three-week study period, no participant received concurrent rehabilitation therapies such as physiotherapy, occupational therapy, or pharmacological gait-modifying agents outside the protocolized orthosis-training sessions; this was confirmed by participant self-report at each visit. The deliberately short three-week study window further minimized the likelihood of meaningful changes in non-protocolized care during the protocol.

### 2.5. Outcome Measures

All assessments were performed at two time points, baseline (Visit 2, prior to the first training session) and post intervention (Visit 7, after completion of all six sessions), by trained evaluators not involved in training delivery.

#### 2.5.1. Primary Outcomes: Walking Performance

The Six-Minute Walk Test (6MWT) measured total distance walked in six minutes as an index of walking endurance [23]. The Ten-Meter Walk Test (10MWT) assessed gait speed (m/s) and walking time (seconds) at a comfortable self-selected pace [24]. Both tests were administered under two conditions: without the device (WO condition) to assess unassisted functional ambulation, and with the powered orthosis (WITH condition) to assess device-assisted performance.

#### 2.5.2. Secondary Outcomes: Balance and Functional Mobility

The Berg Balance Scale (BBS; range 0 to 56) evaluates static and dynamic balance across 14 functional tasks, with excellent reliability (intraclass correlation coefficient [ICC] greater than 0.95) and validity in neurological populations [25,26]. The Timed Up and Go Test (TUG) measures the time required to rise from a chair, walk three meters, turn, return, and sit, with excellent inter-rater and test–retest reliability [27,28]. The Dynamic Gait Index (DGI; range 0 to 24) evaluates the ability to adapt gait to complex walking conditions, with high reliability and concurrent validity in stroke [29]. All secondary outcomes were assessed without the device.

### 2.6. Statistical Analysis

Descriptive statistics characterized demographic data and baseline measurements. Continuous variables are presented as the median and interquartile range (IQR), given the non-normal distributions confirmed by the Shapiro–Wilk test; normally distributed variables are presented as the mean and standard deviation (SD). Within-group pre-to-post comparisons were performed using the Wilcoxon signed-rank test. Effect size was calculated as r = |Z|/√N, where r ≥ 0.1, ≥0.3, and ≥0.5 indicate small, medium, and large effects, respectively. A two-tailed *p*-value less than 0.05 was considered statistically significant. Given the nonrandomized allocation design and marked baseline differences between groups, no between-group comparisons were performed; findings are reported as within-group pre-to-post changes only. Analyses were performed using SPSS (version 22.0; IBM Corp., Armonk, NY, USA) and R (version 4.1.3; R Foundation for Statistical Computing, Vienna, Austria). Complete statistical output, including Z statistics and both exact and asymptotic *p* values for all pre-to-post comparisons, is provided in Supplementary Table S1.

## 3. Results

### 3.1. Participant Characteristics

A total of 75 participants were enrolled and allocated: 39 to the HO group and 36 to the KAFO group. Five participants in the KAFO group were lost to follow-up after allocation (Figure 1) due to personal scheduling conflicts unrelated to the study intervention; none of these participants reported device-related concerns, discomfort, or treatment burden as a reason for withdrawal. This resulted in 31 participants completing post-training assessments in the KAFO group, while all 39 HO participants completed the full protocol. Clinical scale assessments (BBS, TUG, DGI) were completed by 31 participants in the KAFO group and all 39 participants in the HO group at post-training. Gait performance measures under the WO and WITH conditions were available for 31 participants in the KAFO group and 39 in the HO group. Baseline lower extremity muscle strength assessed by the MMT was comparable between the two groups across all major joint levels (hip, knee, and ankle), with median scores ranging from 3.33 to 4 in both groups (Table 1). Baseline demographic and clinical characteristics are presented in Table 1.

### 3.2. Primary Outcomes: Changes in Walking Performance

Pre- to post-training changes in 6MWT and 10MWT under WO and WITH conditions are presented in Table 2. Both groups showed significant improvements across most conditions, with one exception noted below for the KAFO group.

#### 3.2.1. KAFO Group

Unassisted 6MWT distance increased from 205.5 to 227.7 m (*n* = 31, *p* < 0.001, r = 0.668) and 10MWT speed improved from 0.67 to 0.76 m/s (*n* = 31, *p* = 0.007, r = 0.483). Under the WITH condition, 6MWT distance showed a small but statistically significant decrease (237.1 to 233.0 m, *n* = 31, *p* = 0.001, r = 0.601; absolute change −4.1 m), which fell well below established minimal clinically important difference (MCID) thresholds for 6MWT in stroke populations (approximately 34–50 m) and should therefore be regarded as statistically detectable but not clinically meaningful. In contrast, device-assisted 10MWT speed improved from 0.63 to 0.72 m/s (*n* = 31, *p* < 0.001, r = 0.806).

#### 3.2.2. HO Group

Unassisted 6MWT distance increased from 300.0 to 331.5 m (*n* = 39, *p* = 0.001, r = 0.511) and 10MWT speed improved from 1.25 to 1.36 m/s (*n* = 39, *p* < 0.001, r = 0.559). Device-assisted improvements were also significant: 6MWT increased from 275.0 to 304.2 m (*n* = 39, *p* < 0.001, r = 0.679) and 10MWT speed improved from 1.00 to 1.19 m/s (*n* = 39, *p* < 0.001, r = 0.835).

### 3.3. Comparison of Without-Device and With-Device Conditions

Table 3 presents WO versus WITH comparisons at baseline and post-training, based on participants who completed both conditions at each time point (KAFO *n* = 31; HO *n* = 39). In the KAFO group at baseline, unassisted walking was significantly faster than device-assisted walking (10MWT speed: 0.67 versus 0.63 m/s, *p* < 0.001; time: 7.44 versus 7.88 s, *p* < 0.001), reflecting the initial mechanical burden imposed by the device. After training, this gap narrowed substantially; 10MWT time no longer differed significantly between conditions (6.57 versus 6.95 s, *p* = 0.116), while 10MWT speed (*p* = 0.011) and 6MWT distance (*p* = 0.022) differences persisted but were attenuated.

In the HO group, unassisted walking was consistently faster than device-assisted walking at both time points (baseline 6MWT: 300.0 versus 275.0 m, *p* < 0.001; post-training 6MWT: 331.5 versus 304.2 m, *p* < 0.001). Importantly, both conditions improved significantly from pre- to post-training (Table 2), indicating that training benefits transferred to both assisted and unassisted ambulation.

### 3.4. Secondary Outcomes: Changes in Balance and Functional Mobility

Pre- to post-training changes in the BBS, TUG, and DGI are presented in Table 4. In the KAFO group (*n* = 31), all three measures improved significantly. BBS increased by a median of 5.5 points (43.5 to 49.0, *p* = 0.0005, r = 0.625), TUG time decreased by 5.1 s (17.9 to 12.8 s, *p* < 0.001, r = 0.679), and DGI improved by 6.0 points (16.0 to 22.0, *p* = 0.002, r = 0.567). In the HO group (*n* = 39), all three measures showed statistically significant pre-to-post differences with large effect sizes, but the absolute magnitudes of change were small. The BBS increased from a median of 50.0 to 51.0 (Δ +1.0 point, *p* < 0.001, r = 0.633), TUG time increased from 12.6 to 13.4 s (Δ +0.8 s, *p* < 0.001, r = 0.685), and the DGI decreased from 19.0 to 17.0 (Δ −2.0 points, *p* < 0.001, r = 0.633). Each of these changes fell at or below published minimum detectable change (MDC) thresholds in stroke and related neurological populations (BBS MDC approximately 5 points; TUG MDC approximately 3.5–8 s; DGI MDC approximately 2.9 points), and the directions for the TUG and DGI were opposite to a training effect. Consistent with the high baseline functional level of the HO group (median BBS 50.0 of 56; DGI 19.0 of 24), these findings are interpreted cautiously as statistically detectable but clinically non-meaningful fluctuations rather than evidence of functional improvement or deterioration, as discussed further below.

### 3.5. Safety and Adverse Events

No adverse events were observed or reported during the training sessions or post-training assessment period in either group. No falls, near-falls, skin breakdown, device-related discomfort requiring session termination, or other clinically significant adverse symptoms were documented in the standardized case-report forms across the 75 enrolled participants and 450 total training sessions delivered (39 × 6 = 234 in the HO group; 36 × 6 = 216 in the KAFO group, including the five participants subsequently lost to follow-up). All participants tolerated the assigned protocol without incident.

## 4. Discussion

This multicenter prospective pilot study characterized the within-group short-term changes associated with six sessions of overground powered gait orthosis training in patients with neurological gait impairment allocated to two device groups based on clinical gait pattern assessment. The principal findings are as follows. Both groups demonstrated significant improvements in primary walking outcomes under WO and WITH conditions following the three-week training program. Differential response patterns were observed in a manner broadly consistent with each device’s biomechanical target: the KAFO group showed clinically meaningful gains in balance and functional mobility, whereas the HO group demonstrated greater absolute gains in walking speed and endurance. These findings, together with the narrowing of the WO-WITH performance gap in the KAFO group after training, support the clinical plausibility of a gait pattern-matched approach to powered orthosis prescription. However, given the nonrandomized allocation and marked baseline differences between groups, these observations cannot be interpreted as evidence of comparative effectiveness between devices.

The improvements in unassisted walking performance observed in both groups are consistent with the growing body of evidence supporting overground wearable exoskeleton-assisted gait training in neurological populations. A systematic review and meta-analysis by Hsu et al. reported that exoskeleton-assisted training was superior to dose-matched conventional gait training for walking speed (mean difference 0.13 m/s) and balance at the end of intervention [30]. A further systematic review focusing on chronic stroke patients similarly confirmed improvements in gait speed and endurance following wearable exoskeleton training [9]. Our findings extend this literature by demonstrating functionally relevant gains after only six sessions, suggesting that brief, impairment-targeted training can produce meaningful outcomes without the extended schedules required by treadmill-based systems.

In the HO group, 10MWT speed improved by a median of 0.11 m/s in the WO condition, which exceeds commonly cited minimal clinically important difference (MCID) thresholds for gait speed in neurological populations (approximately 0.06–0.10 m/s) [31]. Lee et al. reported a comparable magnitude of walking speed improvement using a similar hip-assist robot in a pilot randomized controlled trial of chronic stroke patients, supporting the generalizability of hip-focused powered assistance for improving gait efficiency [17]. In the KAFO group, WO 10MWT speed improved by 0.09 m/s and was accompanied by clinically meaningful gains in the BBS (+5.5 points), TUG, and DGI (+6.0 points) whose magnitudes exceeded published minimum detectable change thresholds. These BBS and DGI changes exceeded minimum detectable change thresholds of 4 to 6 points reported for stroke patients [26,32], indicating genuine clinical change rather than measurement variability. The magnitude of 6MWT improvement in both groups (KAFO: +22.2 m WO; HO: +31.5 m WO) compares favorably with other wearable exoskeleton studies. Molteni et al. reported a mean 6MWT improvement of approximately 47 m in subacute stroke patients after 15 sessions of EksoGT training [33], and Chang et al., in a large international multicenter randomized controlled trial of subacute stroke patients, reported that a torque-assisted exoskeleton was not superior to dose-matched conventional gait training for ambulatory recovery, although it provided additional gains in lower extremity strength [34]. Our findings suggest that impairment-targeted, shorter-term training may achieve comparable improvements with fewer sessions. Importantly, whereas prior exoskeleton studies have predominantly evaluated a single device type in homogeneous diagnostic populations, the present study is the first multicenter study to prospectively allocate patients to two distinct powered orthosis types based on individual gait pattern assessment, thereby directly addressing a clinically relevant gap in the prescription framework for wearable gait orthoses.

A key finding is the divergence in secondary outcome profiles between the two groups. In the KAFO group, the BBS, TUG, and DGI all improved, with magnitudes exceeding published MDC thresholds, indicating clinically meaningful gains in balance and functional mobility. In the HO group, by contrast, all three measures showed pre-to-post differences that were statistically significant with large effect sizes (all *p* < 0.001; r > 0.6), yet the absolute magnitudes of change were small: BBS +1.0 point, TUG +0.8 s, and DGI −2.0 points. Interpretation of this HO pattern requires careful separation of three distinct concepts: statistical significance, effect size, and clinical meaningfulness. The low *p*-values and large effect sizes indicate that the paired differences across participants were directionally consistent, but the Wilcoxon-based effect size r reflects the consistency of within-pair change direction rather than the magnitude of that change. Accordingly, all three HO changes fell at or below published minimum detectable change thresholds in stroke and related neurological populations (BBS MDC approximately 5 points [26]; TUG MDC reported in the 3.5–8 s range in chronic stroke and Parkinson’s disease populations; DGI MDC approximately 2.9 points [32]), and the TUG change was also well below the reported MCID for subacute stroke (approximately 3.4 s). These observations argue against genuinely clinically relevant change in any direction, and are more parsimoniously explained by the high baseline functional level of the HO group (median BBS 50.0 of 56; TUG 12.6 s; DGI 19.0 of 24), which left limited headroom for measurable improvement while preserving scope for small bidirectional fluctuations around the personal baseline. Additional non-random contributors may include residual neuromuscular fatigue or attentional load at the time of unassisted post-training reassessment, which followed cumulative device-assisted walking practice and could plausibly contribute small decrements on tasks sensitive to cognitive–motor integration such as the DGI, and assessor variability across the four participating centers, which despite standardization cannot be fully excluded. Given the non-randomized design and the absence of a no-training control, these HO secondary-outcome findings should be interpreted as hypothesis-generating and warrant verification in a larger randomized study with blinded assessors and a priori powered secondary outcomes. The two groups differed considerably in baseline walking performance, diagnostic composition, and sex ratio, reflecting the nature of clinical allocation rather than randomization. The observed differential response patterns should therefore be interpreted as within-group changes specific to each device–pathway pairing, not as evidence of comparative device efficacy.

These differential response patterns are biomechanically explicable. The KAFO addresses knee and ankle weakness by actively generating swing-phase knee flexion, thereby eliminating pathological compensatory gait strategies such as hip circumduction, trunk lean, and vaulting that characterize walking in patients with knee extensor weakness and foot drop [35]. Repeated, correctly patterned knee flexion–extension cycles during training are likely to facilitate motor re-learning, proprioceptive input, and activation of paretic quadriceps and tibialis anterior muscles, consistent with principles of task-specific repetitive gait training [36]. In contrast, the HO targets hip flexion and extension, producing improvements primarily in stride length, walking speed, and endurance through enhanced hip propulsion rather than through balance retraining.

The comparison between WO and WITH conditions provides additional insight into how device effects evolve with training. In the KAFO group at baseline, device-assisted 10MWT speed (0.63 m/s) was lower than unassisted speed (0.67 m/s), reflecting the added mechanical load during the initial adaptation phase [31]. After six sessions, the WO-WITH gap narrowed substantially and 10MWT time no longer differed significantly between conditions (*p* = 0.116), suggesting that patients increasingly integrated KAFO assistance as motor learning progressed [21]. An alternative, complementary interpretation is that as KAFO participants’ unassisted walking capacity improved with training, the relative mechanical load of carrying the device may have become a more salient constraint on endurance performance, narrowing the margin by which device-assisted endurance could exceed unassisted endurance. This hypothesis, namely that training shifts the physical-effort calculus in favor of unassisted gait, is consistent with the convergence of WO and WITH 10MWT times after training and warrants direct investigation in future studies incorporating metabolic cost measurements such as oxygen consumption or perceived exertion ratings. Notably, device-assisted 6MWT distance in the KAFO group showed a small statistically significant decrease (237.1 to 233.0 m, *p* = 0.001; absolute change −4.1 m), in contrast to the significant +22.2 m gain observed in the WO condition. However, the magnitude of this decrease is well below reported minimal clinically important difference thresholds for the 6MWT in stroke (approximately 34–50 m) and likely reflects measurement variability rather than true functional deterioration. Taken together with the concurrent WITH-condition improvement in 10MWT speed, this pattern suggests that short-burst speed tasks benefited from device-assisted training while endurance performance under device load remained essentially unchanged. One plausible explanation is that the sustained metabolic and biomechanical burden of the KAFO over a prolonged walking distance may continue to constrain endurance even as short-distance mechanics improve with adaptation. This finding underscores that device-assisted endurance capacity may require a longer training period to show meaningful gains and warrants attention in future dosing studies. In the HO group, WO performance consistently exceeded WITH performance at both time points, which is mechanistically expected given the higher baseline ambulatory ability of this group and the bilateral actuator load. Despite this, both conditions improved significantly, confirming that training benefits transferred to unassisted ambulation [17].

Several limitations should be acknowledged. First, the absence of a non-intervention control group precludes definitive attribution of observed pre-to-post changes to the orthosis training intervention. Natural neurological recovery during the study window, repeated test exposure, and any unmeasured concurrent care may have contributed to the observed changes; this is particularly relevant for participants in earlier post-injury phases. Although no participant initiated new rehabilitation therapies during the three-week study window, the study window does not entirely preclude these confounders. Second, the nonrandomized clinical allocation and marked baseline imbalance between groups (including differences in baseline gait performance, diagnostic composition, and sex ratio) preclude any comparative interpretation of outcome differences across groups; all findings are reported as within-group changes only. Third, the clinical allocation criteria, while operationally defined, were not algorithmically standardized across sites, and no formal inter-rater calibration was conducted; this may have introduced inconsistency in device assignment. Fourth, five participants in the KAFO group were lost to follow-up after allocation. Comparison of baseline characteristics between KAFO completers (*n* = 31) and these participants was not feasible because complete baseline data for the dropout subgroup were not retained beyond the allocation visit. Analyses were therefore conducted as complete-case analyses without imputation; no formal sensitivity or intention-to-treat analysis was performed, and residual attrition bias cannot be entirely excluded. Fifth, the six-session protocol is deliberately brief; optimal dosing and longer-term effects remain to be determined. Sixth, the absence of post-training follow-up prevents conclusions about the durability of improvement. Finally, the absence of blinding and the involvement of device manufacturer-affiliated authors in data collection, though not in analysis, introduces potential performance and assessment bias. Additionally, the MCID and MDC thresholds used to interpret the magnitude of observed changes were drawn from published reports in stroke and mixed neurological populations. Because our sample included mixed neurological diagnoses (predominantly stroke but also other central nervous system disorders), these thresholds were used descriptively as comparative benchmarks rather than as formal hypothesis-testing criteria, and their applicability to non-stroke participants cannot be assumed. This descriptive use is acknowledged as a limitation of cross-population threshold transferability.

Future studies should prioritize a prospective randomized controlled trial with a conventional gait training comparator arm, blinded outcome assessment, and long-term follow-up. Biomechanical analyses including kinematic and electromyographic assessment would clarify the mechanisms by which each device facilitates motor learning. Development of a validated clinical protocol for individualized orthosis prescription represents an important translational priority. Looking forward, hybrid neurorehabilitation paradigms that combine powered orthoses with complementary modalities such as functional electrical stimulation (FES) and smart-textile electrode systems may offer pathways to address some of the limitations encountered in the present study. Recent feasibility work with AI-driven, textile-integrated multichannel FES systems has demonstrated that synchronized, phase-specific electrical stimulation can be safely delivered through wearable garments in post-stroke gait recovery [37] and in chronic neurological gait impairments of mixed etiology [38]. In principle, hybrid mechanical–FES configurations could mitigate the metabolic cost imposed by the actuators and reducers of powered orthoses while preserving the kinematic guidance these devices provide, potentially narrowing the WO-WITH performance gap more effectively than mechanical assistance alone. Although direct comparative evidence is not yet available, hybrid mechanical–FES architectures are an active area of development and represent a logical next step in the personalization of impairment-targeted gait training.

## 5. Conclusions

In this multicenter prospective pilot study, six sessions of overground powered gait orthosis training over three weeks produced clinically meaningful within-group improvements in walking outcomes in both groups. In the KAFO group, balance and functional mobility outcomes also improved with magnitudes that exceeded published minimum detectable change thresholds, whereas in the HO group balance and functional mobility outcomes showed only statistically detectable but clinically non-meaningful fluctuations around near-ceiling baseline scores. Walking benefits generalized to unassisted ambulation in both groups, and the within-group response patterns were broadly consistent with each device’s biomechanical target. However, given the nonrandomized clinical allocation and marked baseline differences between groups, these findings should not be interpreted as comparative effectiveness evidence between the two devices. Rather, they demonstrate the feasibility of a gait pattern-matched approach to powered orthosis prescription and provide the observational basis for future randomized controlled trials evaluating pattern-matched gait training against conventional rehabilitation.

## Figures and Tables

**Table 1:** Baseline demographic and clinical characteristics.

Variable	HO (*n* = 39)	KAFO (*n* = 36)
Age, years	65.4 ± 9.2	60.3 ± 12.3
Sex, male/female	19/20	29/7
BMI, kg/m^2^	24.7 ± 3.2	25.4 ± 3.4
FAC score	3.0 [2–4]	2.5 [2–4]
Diagnosis, n (%)		
Stroke	22 (56.4)	32 (88.9)
Traumatic brain injury	2 (5.1)	1 (2.8)
Other neurological (e.g., cerebral palsy, spinal cord injury, Parkinson’s disease)	15 (38.5)	3 (8.3)
Baseline outcomes, median [IQR]		
BBS, score	50.0 [41.0–53.0]	43.5 [36.0–47.8]
TUG, s	12.6 [10.0–18.5]	17.9 [14.3–27.8]
DGI, score	19.0 [12.0–23.0]	16.0 [13.3–21.8]
6MWT (WO), m	300.0 [242.9–413.6]	205.5 [142.5–274.5]
10MWT speed (WO), m/s	1.25 [0.79–1.51]	0.67 [0.49–0.84]
MMT, median [IQR]		
Hip flexion	4 [3–4]	4 [3–4.33]
Hip extension	3.33 [3–4]	3.33 [3–4]
Knee flexion	3.33 [3–4]	3.67 [3–4.33]
Knee extension	4 [4–5]	4 [3.33–5]
Ankle dorsiflexion	4 [2–4.83]	4 [3–4.33]
Ankle plantarflexion	4 [2.08–4.83]	4 [3–4.33]

Values are mean ± SD, *n* (%), or median [IQR]. FAC = Functional Ambulation Category; BBS = Berg Balance Scale; TUG = Timed Up and Go Test; DGI = Dynamic Gait Index; 6MWT = Six-Minute Walk Test; 10MWT = Ten-Meter Walk Test; WO = without device; MMT = Manual Muscle Testing; IQR = interquartile range.

**Table 2:** Changes in walking performance after training under without-device (WO) and with-device (WITH) conditions.

Outcome	Condition	*n*	Pre-Training Median [IQR]	Post-Training Median [IQR]	*p*-Value	r
KAFO group						
6MWT (m)	WO	31	205.5 [142.5–274.5]	227.7 [168.4–323.6]	<0.001	0.668
	WITH	31	237.1 [191.1–280.7]	233.0 [160.6–308.6]	0.001	0.601
10MWT speed (m/s)	WO	31	0.67 [0.49–0.84]	0.76 [0.51–1.05]	0.007	0.483
	WITH	31	0.63 [0.40–0.74]	0.72 [0.51–0.91]	<0.001	0.806
10MWT time (s)	WO	31	7.44 [5.93–10.27]	6.57 [4.76–9.73]	0.035	0.378
	WITH	31	7.88 [6.74–12.35]	6.95 [5.46–9.75]	<0.001	0.676
HO group						
6MWT (m)	WO	39	300.0 [242.9–413.6]	331.5 [269.5–422.7]	0.001	0.511
	WITH	39	275.0 [198.6–333.8]	304.2 [245.1–362.4]	<0.001	0.679
10MWT speed (m/s)	WO	39	1.25 [0.79–1.51]	1.36 [0.96–1.60]	<0.001	0.559
	WITH	39	1.00 [0.70–1.24]	1.19 [0.98–1.41]	<0.001	0.835
10MWT time (s)	WO	39	4.01 [3.32–6.31]	3.68 [3.13–5.19]	<0.001	0.619
	WITH	39	4.97 [4.05–7.09]	4.20 [3.55–5.10]	<0.001	0.836

*n* = number of participants with complete paired pre- and post-training data for each condition. r = effect size (|Z|/√N); r ≥ 0.1 small, ≥0.3 medium, ≥0.5 large. 6MWT = Six-Minute Walk Test; 10MWT = Ten-Meter Walk Test; WO = without device; WITH = with device; IQR = interquartile range.

**Table 3:** Comparison of without-device (WO) and with-device (WITH) walking performance at baseline and post-training.

Outcome	*n*	Without Device (WO) Median [IQR]	With Device (WITH) Median [IQR]	*p*-Value
KAFO group—Baseline				
6MWT (m)	31	205.5 [142.5–274.5]	237.1 [191.1–280.7]	0.003
10MWT speed (m/s)	31	0.67 [0.49–0.84]	0.63 [0.40–0.74]	<0.001
10MWT time (s)	31	7.44 [5.93–10.27]	7.88 [6.74–12.35]	<0.001
KAFO group—Post-training				
6MWT (m)	31	227.7 [168.4–323.6]	233.0 [160.6–308.6]	0.022
10MWT speed (m/s)	31	0.76 [0.51–1.05]	0.72 [0.51–0.91]	0.011
10MWT time (s)	31	6.57 [4.76–9.73]	6.95 [5.46–9.75]	0.116
HO group—Baseline				
6MWT (m)	39	300.0 [242.9–413.6]	275.0 [198.6–333.8]	<0.001
10MWT speed (m/s)	39	1.25 [0.79–1.51]	1.00 [0.70–1.24]	<0.001
10MWT time (s)	39	4.01 [3.32–6.31]	4.97 [4.05–7.09]	<0.001
HO group—Post-training				
6MWT (m)	39	331.5 [269.5–422.7]	304.2 [245.1–362.4]	<0.001
10MWT speed (m/s)	39	1.36 [0.96–1.60]	1.19 [0.98–1.41]	<0.001
10MWT time (s)	39	3.68 [3.13–5.19]	4.20 [3.55–5.10]	0.002

*n* = number of participants with complete paired WO and WITH data at each time point. 6MWT = Six-Minute Walk Test; 10MWT = Ten-Meter Walk Test; WO = without device; WITH = with device; IQR = interquartile range.

**Table 4:** Changes in balance and functional mobility after training.

Outcome	*n*	Pre-Training Median [IQR]	Post-Training Median [IQR]	Δ Median	*p*-Value	r
KAFO group						
BBS (score)	31	43.5 [36.0–47.8]	49.0 [46.0–53.0]	+5.5	0.0005	0.625
TUG (s)	31	17.9 [14.3–27.8]	12.8 [9.7–14.5]	−5.1	<0.001	0.679
DGI (score)	31	16.0 [13.3–21.8]	22.0 [16.0–23.0]	+6.0	0.002	0.567
HO group						
BBS (score)	39	50.0 [41.0–53.0]	51.0 [40.0–53.0]	+1.0	<0.001	0.633
TUG (s)	39	12.6 [10.0–18.5]	13.4 [8.8–15.7]	+0.8	<0.001	0.685
DGI (score)	39	19.0 [12.0–23.0]	17.0 [13.0–23.0]	−2.0	<0.001	0.633

*n* = number of participants with complete paired pre- and post-training data. r = effect size (|Z|/√N); r ≥ 0.1 small, ≥0.3 medium, ≥0.5 large. BBS = Berg Balance Scale; TUG = Timed Up and Go Test; DGI = Dynamic Gait Index; IQR = interquartile range; Δ Median = post-training minus pre-training median.

## Data Availability

The data presented in this study are available on request from the corresponding author.

## Supplementary Materials

The following supporting information can be downloaded at: https://www.mdpi.com/article/10.3390/jcm15103580/s1, Table S1: Complete statistical output for within-group pre-to-post comparisons (Wilcoxon signed-rank tests).
