# Supplementary material for: Pattern-Matched Powered Gait Orthosis Training in Patients with Neurological Gait Impairment: A Multicenter Prospective Pilot Study of Hip and Knee–Ankle–Foot Orthoses
Source: J Clin Med. 2026 May 7;15(10):3580. doi: 10.3390/jcm15103580 (PMC13207410; doi:10.3390/jcm15103580)
Supplement: Supplementary file 1 [file jcm-15-03580-s001.zip › jcm-4305453-supplementary/Supplementary_Material.pdf]

Supplementary Materials

Table S1. Complete statistical output for within-group pre-to-post comparisons (Wilcoxon signed-rank tests).

Outcome	n paired	Pre-training			Post-training			Z	Exact p (2-tailed)	Asymp. p (2-tailed)	r
		Median	25th	75th	Median	25th	75th				
**Hip Orthosis (HO) group (n = 39)**											
6MWT WO (m)	39	300.00	242.90	413.58	331.45	269.50	422.68	−3.192	$9.93×10^{-4}$	$1.42×10^{-3}$	0.511
6MWT WITH (m)	39	275.01	198.63	333.75	304.20	245.05	362.38	−4.240	$4.32×10^{-5}$	$2.24×10^{-5}$	0.688
10MWT WO (m/s)	39	1.247	0.792	1.506	1.358	0.960	1.596	−3.489	$2.84×10^{-4}$	$4.85×10^{-4}$	0.559
10MWT WITH (m/s)	39	1.000	0.700	1.235	1.190	0.980	1.410	−5.212	$<1.0×10^{-6}$	$1.86×10^{-7}$	0.834
BBS (score)	39	50.0	41.0	53.0	51.0	40.0	53.0	−3.954	$3.81×10^{-6}$	$7.69×10^{-5}$	0.633
TUG (s)	39	12.56	9.99	18.51	13.36	8.82	15.68	−4.276	$8.20×10^{-7}$	$1.90×10^{-5}$	0.685
DGI (score)	39	19.0	12.0	23.0	17.0	13.0	23.0	−3.950	$1.91×10^{-6}$	$7.82×10^{-5}$	0.633
**Knee-Ankle-Foot Orthosis (KAFO) group (n = 31 paired after 5 dropouts)**											
6MWT WO (m)	31	205.50	142.50	274.50	227.70	168.40	323.58	−3.708	$8.43×10^{-5}$	$2.01×10^{-4}$	0.666
6MWT WITH (m)	31	237.10	191.10	280.70	232.95	160.65	308.58	−3.346	$4.19×10^{-4}$	$8.21×10^{-4}$	0.601
10MWT WO (m/s)	31	0.674	0.487	0.844	0.760	0.514	1.045	−2.687	$6.32×10^{-3}$	$7.22×10^{-3}$	0.483
10MWT WITH (m/s)	31	0.630	0.405	0.742	0.716	0.513	0.908	−4.488	$3.05×10^{-6}$	$7.18×10^{-6}$	0.806
BBS (score)	31	43.5	36.0	47.75	49.0	46.0	53.0	−3.482	$6.10×10^{-5}$	$4.98×10^{-4}$	0.625
TUG (s)	31	17.89	14.34	27.77	12.79	9.74	14.53	−3.783	$7.63×10^{-6}$	$1.55×10^{-4}$	0.679
DGI (score)	31	16.0	13.25	21.75	22.0	16.0	23.0	−3.156	$6.10×10^{-4}$	$1.60×10^{-3}$	0.567

Notes. Wilcoxon signed-rank test was used for all within-group pre-to-post comparisons. Effect size $r = |Z| / \sqrt{n}$, where n is the number of paired observations. Effect size thresholds: $r \geq 0.1$ (small), $r \geq 0.3$ (medium), $r \geq 0.5$ (large). Exact p values are preferred for smaller samples; asymptotic p values are provided for reference. Both are two-tailed.

Abbreviations: 6MWT = Six-Minute Walk Test; 10MWT = Ten-Meter Walk Test; BBS = Berg Balance Scale; TUG = Timed Up and Go Test; DGI = Dynamic Gait Index; WO = without device; WITH = with device.
